# Multilevel analysis of individual and community level factors associated with institutional delivery in Ethiopia

**DOI:** 10.1186/s13104-015-1343-1

**Published:** 2015-08-26

**Authors:** Zeleke A. Mekonnen, Wondwossen T. Lerebo, Tesfay G. Gebrehiwot, Samir A. Abadura

**Affiliations:** Health Promotion and Disease Prevention Directorate, Federal Ministry of Health, P.O. Box. 1234, Addis Ababa, Ethiopia; Department of Public Health, Mekelle University, Mekelle, Ethiopia; Tulane International, Addis Ababa, Ethiopia; Oromia Regional Health Bureau, Addis Ababa, Ethiopia

**Keywords:** Institutional delivery, EDHS, Community, Multilevel analysis, Ethiopia

## Abstract

**Background:**

Improving maternal health is one of the eight millennium development goals to reduce maternal mortality (MM) by three quarters between 1990 and 2015. Institutional delivery is considered to be the most critical intervention in reducing MM and ensuring safe motherhood. However, the level of maternal morbidity and mortality in Ethiopia are among the highest in the world and the proportion of births occurring at health facilities is very low. This study examined the individual and community level factors associated with institutional delivery in Ethiopia.

**Methods:**

Data from the 2011 Ethiopian demographic and health survey were used to identify individual and community level factors associated with institutional delivery among women who had a live birth during the 5 years preceding the survey. Taking into account the nested structure of the data, multilevel logistic regression analysis has been employed to a nationally representative sample of 7757 women nested with in 595 communities.

**Results:**

At the individual level; higher educational level of the women (AOR = 3.60; 95 % CI 2.491–5.214), women from richest households (AOR = 1.74; 95 % CI 1.143–2.648) and increased ante natal care attendance (AOR = 4.43; 95 % CI 3.405–5.751) were associated with institutional delivery. Additionally, at the community level; urban residence (AOR = 4.74; 95 % CI 3.196–7.039), residing in communities with high proportion of educated women (AOR = 1.71; 95 % CI 1.256–2.319) and residing in communities with high ANC utilization rate (AOR = 1.55; 95 % CI 1.132–2.127) had a significant effect on institutional delivery. Also region and distance to health facility showed significant association with institutional delivery. The random effects showed that the variation in institutional delivery service utilization between communities was statistically significant.

**Conclusion:**

Both individual and community level factors are associated with institutional delivery service uptake. As a result, further research is needed to better understand why these factors may affect institutional delivery.

## Background

Every day women die from pregnancy and birth related complications. Globally, in 2010 there were 287,000 maternal deaths of which developing countries account for 99 % with Sub Saharan Africa alone accounting for 56 %. Maternal mortality (MM) remains unacceptably high and has become major challenge in most developing countries including Ethiopia (MM of 676/100,000 live births) where maternal mortality and morbidity levels are among the highest in the world [1, 2].

About 80 % of maternal deaths are due to causes directly related to pregnancy and child birth. The major causes of maternal deaths are hemorrhage, infections, unsafe abortion, obstructed labour and hypertension during pregnancy. Though many of these complications are unpredictable, almost all could have been prevented by ensuring institutional delivery services as timely management and treatment can make the difference between life and death [3–5].

Improving maternal health requires increasing the proportion of mothers who are giving birth at health institutions and attended by skilled health workers. The statistics revealed that nearly all births in developed countries, 61.9 % in less developed countries, 46.9 % in south central Asia and 33.7 % in eastern Africa were attended by skilled health workers [6]. In Ethiopia, despite the progress that has been made to improve maternal and child health, the proportion of births occurring at health institutions is still very low (10 %) [7] and it has remained as an unaddressed top priority of the country [8, 9].

Research conducted in different countries shows that various socio economic, demographic, physical accessibility and community related factors influence the women’s decision to use institutional delivery services [10–17]. In Ethiopia prior studies have been done to identify the socio-economic and demographic factors that influence institutional delivery service utilization [18–22]. Though institutional delivery services are affected by factors operating at different levels including the community contextual effects [11, 15–17, 23–25], none of the studies have tried to look at the factors that affect institutional delivery service utilization at individual and community levels simultaneously.

Hence, this study aimed to examine the individual and community level factors associated with institutional delivery simultaneously with the application of multilevel modeling and provide evidence for policy makers to better understand potential factors affecting institutional delivery.

## Methods

### Data source

The analysis was based on the 2011 Ethiopian demographic and health survey (EDHS) data. Approval letter for the use of this data was gained from the Measure DHS and the data set was downloaded from the Measure DHS website www.meauredhs.com. The survey covered all the nine regions and two city administrations of Ethiopia and participants were selected through a stratified two stage cluster sampling technique. The full details of the methods and procedures used in data collection in the EDHS have been published elsewhere [7]. The survey collected information from a nationally representative sample of 16,515 women aged 15–49 years. The study populations for this study were 7757 women who had at least one live birth in the 5 years preceding the survey, nested with in 595 communities across the country.

### Study variables

#### Outcome variable

The main outcome variable in this study was whether a women had institutional delivery for the most recent live birth or not. It is a binary variable categorized as Yes or No.

#### Explanatory variables

These are individual level factors (age group of women, marital status, religion, women education, and husband education, sex of house hold head, health care decision, household wealth index, media exposure, birth order and ANC visit) and community level factors (region, place of residence, distance to health facility, community poverty, community women’s education, community media exposure and Community ANC utilization rate). The aggregate community level explanatory variables were constructed by aggregating individual level characteristics at the community (cluster) level and categorization of the aggregate variables was done as high or low based on the distribution of the proportion values calculated for each community. Histogram was used to check the distribution of the proportion values. If the aggregate variable was normally distributed mean value and if not normally distributed median value was used as cut off point for the categorization (*Community poverty* was categorized as high if the proportion of women from the two lowest wealth quintiles in a given community was 47–100 % and low if the proportion was 0–46 %, *Community media exposure was* categorized as low if the proportion of women exposed to media in the community was 0–15 % and categorized as high if the proportion was 16–100 %, *Community education* was categorized as low if the proportion of women with secondary education & above in the community was 0 % and categorized as high if the proportion was 1–100 % and *Community ANC utilization rate* was categorised as low if the proportion of women who attended at least one ANC visit in the community was 0–45 % and categorized as high if the proportion was between 46 and 100 %.

### Data analysis

A multi level logistic regression analysis technique was employed in this study in order to account for the hierarchal structure of the DHS data and the binary response of the outcome variable [26–28].

Bivariate multilevel logistic regression analysis was performed to estimate the crude odds ratios at 95 % confidence interval and those variables which were statistically significant were considered in the multivariate analysis. Finally, multivariate multilevel logistic regression analysis was performed to estimate the adjusted odds ratios and to estimate the extent of random variations between communities.

#### Model building

Four models containing variables of interest were fitted using the xtmelogit command in STATA version 11.0.

*Model I* (*Empty model*) was fitted without explanatory variables to test random variability in the intercept and to estimate the intra class correlation coefficient (ICC). *Model II* examined the effects of individual level characteristics, *Model III* examined the effect of community level variables and *Model IV* examined the effects of both individual and community level characteristics simultaneously.

The final two level model in which the individual women (level 1) were nested within the community (level 2) was expressed elsewhere [26].

Since the models were nested, the Chi square likelihood-ratio test was used to assess the difference between models. The p-values were estimated using the Wald statistics and a p value <0.05 was considered as statistically significant.

### Parameter estimation methods

In the multilevel models, the fixed effects (measures of association) estimates the association between the likelihood of institutional delivery and the individual and community level factors and were expressed as odds ratio with their 95 % confidence intervals. The random effects are the measures of variation in institutional delivery across communities expressed as ICC and proportional change in variance (PCV). The ICC was calculated to evaluate whether the variation in institutional delivery is primarily within or between communities [29, 30].

### Ethical considerations

Ethical clearance was obtained from the ethical review committee of the college of health sciences of Mekelle University and also approval letter for the use of the EDHS data set was gained from the Measure DHS (ORC MACRO). No information obtained from the data set was disclosed to any third person.

## Results

In this study, a total of 7757 women with their most recent birth in the 5 years prior to the survey were included in the analysis. Among the total women whose data were analyzed 49 % were aged 25–34 years, almost 67 % were uneducated, 29 % were from poorest households and the vast majority (80 %) resides in rural areas (See Tables 1, 2).

**Table 1:** Bivariate analysis of institutional delivery by individual level factors, EDHS 2011

Individual level characteristics	Institutional delivery		Total (%)
	Yes (%)	No (%)	
Age group of women			
15–24 years	396 (19.7)	1616 (80.3)	2012 (25.9)
25–34 years	676 (17.8)	3121 (82.2)	3797 (49.0)
35–49 years	193 (9.9)	1755 (90.1)	1948 (25.1)
Marital status			
Married	1099 (15.6)	5940 (84.4)	7039 (90.7)
Others	166 (23.1)	552 (76.9)	718 (9.3)
Religion			
Orthodox	655 (24.3)	2037 (75.7)	2692 (34.7)
Protestant	171 (11.6)	1306 (88.4)	1477 (19.1)
Muslim	424 (12.6)	2934 (87.4)	3358 (43.3)
Others	15 (6.6)	211 (93.4)	226 (2.9)
Women educational level			
No education	360 (6.9)	4820 (93.1)	5180 (66.8)
Primary	523 (25.0)	1571 (75.0)	2094 (27.0)
Secondary & above	382 (79.1)	101 (20.9)	483 (6.2)
Husband educational level			
No education	213 (5.4)	3715 (94.6)	3928 (51.2)
Primary	484 (17.3)	2306 (82.7)	2790 (36.3)
Secondary & above	538 (55.9)	425 (44.1)	963 (12.5)
Sex of house hold head			
Female	365 (23.4)	1195 (76.6)	1560 (20.1)
Male	900 (14.5)	5297 (85.5)	6197 (79.9)
Health care decision			
Husband	156 (7.9)	1827 (92.1)	1983 (28.2)
Women	299 (27.3)	795 (72.7)	1094 (15.6)
Jointly	644 (16.3)	3309 (83.7)	3953 (56.2)
Household wealth index			
Poorest	85 (3.7)	2192 (96.3)	2277 (29.3)
Poor	55 (4.1)	1299 (95.9)	1354 (17.5)
Middle	49 (3.9)	1190 (96.1)	1239 (16.0)
Rich	111 (9.0)	1118 (91.0)	1229 (15.8)
Richest	965 (58.2)	693 (41.8)	1658 (21.4)
Media exposure			
Has no exposure	516 (8.7)	5419 (91.3)	5935 (76.5)
Has exposure	749 (41.1)	1073 (58.9)	1822 (23.5)
Birth order			
1	519 (35.2)	956 (64.8)	1475 (19.0)
2–3	476 (19.7)	1941 (80.3)	2417 (31.2)
4–5	167 (9.4)	1610 (90.6)	1777 (22.9)
6+	103 (4.9)	1985 (95.1)	2088 (26.9)
ANC visit			
No visit	178 (4.2)	4110 (95.8)	4288 (55.3)
1 visit	31 (9.2)	307 (90.8)	338 (4.4)
2–3 visits	258 (18.1)	1170 (81.9)	1428 (18.4)
4 and above visits	798 (46.9)	905 (53.1)	1703 (21.9)

**Table 2:** Bivariate analysis of institutional delivery by community level factors, EDHS 2011

Community level characteristics	Institutional delivery		Total (%)
	Yes (%)	No (%)	
Region			
Tigray	103 (12.2)	744 (87.8)	847 (10.9)
Affar	41 (5.7)	672 (94.3)	713 (9.2)
Amhara	85 (8.8)	880 (91.2)	965 (12.4)
Oromia	104 (9.5)	996 (90.5)	1100 (14.2)
Somali	50 (8.9)	509 (91.1)	559 (7.2)
Beni-shangul Gumuz	54 (8.0)	619 (92.0)	673 (8.7)
SNNP	69 (6.6)	983 (93.4)	1052 (13.6)
Gambella	113 (18.6)	494 (81.4)	607 (7.8)
Harari	165 (37.5)	275 (62.5)	440 (5.7)
Addis Ababa	288 (83.2)	58 (16.8)	346 (4.5)
Dire Dawa	193 (42.4 %)	262 (57.6)	455 (5.9)
Place of residence			
Rural	322 (5.1)	5925 (94.9)	6247 (80.5)
Urban	943 (62.4)	567 (37.6)	1510 (19.5)
Distance to health facility			
Not big problem	778 (35.5)	1415 (64.5)	2193 (28.3)
Big problem	485 (8.7)	5072 (91.3)	5557 (71.7)
Community poverty			
High	142 (3.6)	3853 (96.4)	3995 (51.5)
Low	1,123 (29.9)	2639 (70.1)	3762 (48.5)
Community women’s education			
Low	311 (5.6)	5204 (94.4)	5515 (71.1)
High	954 (42.6)	1288 (57.4)	2242 (28.9)
Community media exposure			
Low	223 (5.7)	3688 (94.3)	3911 (50.4)
High	1042 (27.1)	2804 (72.9)	3846 (49.6)
Community ANC utilization			
Low	159 (3.6)	4245 (96.4)	4404 (56.8)
High	1106 (33.0)	2247 (67.0)	3353 (43.2)

### Multilevel logistic regression analysis

The fixed effects (measure of association) and the random intercepts for the use of institutional delivery services are presented in Table 3. The results of the empty model (Model I) depicted that there was a statistically significant variability in the odds of institutional delivery service utilization between communities (τ = 10.246, p-value = 0.000). Similarly, the ICC in the empty model implied that 75.6 % of the total variance in the utilization of institutional delivery services was attributed to differences between communities. In Model II only individual level variables were added. The results showed that women education level, husband educational level, household wealth index, media exposure, birth order and antenatal care visits were significantly associated with birth at health institutions. The ICC in Model II indicated that, 25.3 % of the variation in women’s institutional delivery service utilization was attributable to differences across communities. As shown by the PCV, 89.1 % of the variance in institutional delivery service utilization across communities was explained by the individual level characteristics. In Model III only community level variables were added. The result revealed that women from urban areas, residing in communities with low poverty level, residing in communities with high media exposure and women residing in communities with high rate of antenatal care utilization were significantly associated with institutional delivery. The ICC in Model III implied that differences between communities account for about 19 % of the variation in women’s institutional delivery service utilization. In addition, the PCV indicated that 92.4 % of the variation in institutional delivery service utilization between communities was explained by community level characteristics.

**Table 3:** Multilevel logistic regression analysis of individual and community level factors associated with institutional delivery

Characteristics	Model I	Model II	Model III	Model IV
Fixed effects		AOR (95 % CI)	AOR (95 % CI)	AOR (95 % CI)
Women educational level				
No education(R)		1		1
Primary		1.39 (1.107–1.750)	–	1.50 (1.202–1.880)
Secondary & above		2.75 (1.866–4.054)	–	3.60 (2.491–5.214)
Husband educational level				
No education(R)		1		
Primary		1.22 (0.963–1.557)	–	–
Secondary & above		2.10 (1.528–2.883)	–	–
Media exposure				
No exposure (R)		1		1
Has exposure		1.33 (1.065–1.666)	–	1.39 (1.115–1.752)
Household wealth index				
Poorest (R)		1		1
Poor		0.99 (0.664–1.496)	–	1.01 (0.673–1.497)
Middle		0.86 (0.563–1.313)	–	0.75 (0.493–1.137)
Rich		1.49 (1.029–2.183)	–	1.11 (0.765–1.607)
Richest		7.05 (4.862–10.234)	–	1.74 (1.143–2.648)
Birth order				
1 (R)		1		1
2–3		0.56 (0.438–0.709)	–	0.49 (0.385–0.622)
4–5		0.39 (0.289–0.520)	–	0.38 (0.286–0.512)
6+		0.34 (0.247–0.463)	–	0.33 (0.245–0.456)
ANC visit				
No visit (R)		1		1
1 visit		1.97 (1.201–3.229)	–	1.89 (1.148–3.113)
2–3 visits		2.69 (2.058–3.515)	–	2.66 (2.031–3.479)
4 and above		5.09 (3.947–6.574)	–	4.43 (3.405–5.751)
Region				
Affar (R)			1	1
Tigray		–	1.88 (0.948–3.757)	1.56 (0.796–3.072)
Amhara		–	2.46 (1.243–4.872)	3.06 (1.571–5.974)
Oromia		–	1.96 (1.002–3.836)	2.06 (1.065–3.995)
Somali		–	1.87 (0.866–4.062)	2.92 (1.382–6.150)
B/Gumuz		–	2.66 (1.289–5.525)	2.67 (1.310–5.451)
SNNP		–	1.62 (0.800–3.256)	1.61 (0.806–3.218)
Gambella		–	6.76 (3.341–13.698)	5.78 (2.920–11.453)
Harari		–	5.96 (2.953–12.034)	5.52 (2.751–11.066)
Addis Ababa		–	10.65 (5.231–21.686)	6.43 (3.173–13.019)
Dire Dawa		–	9.37 (4.632–18.958)	10.19 (5.088–20.387)
Place of residence				
Rural (R)			1	1
Urban		–	7.46 (5.163–10.777)	4.74 (3.196–7.039)
Distance to health facility				
Not big problem		–	1.66 (1.356–2.032)	1.46 (1.1792–1.805)
Big problem (R)			1	1
Community poverty				
High (R)			1	
Low		–	1.57 (1.115–2.212)	–
Community women’s education				
Low (R)			1	1
High		–	2.66 (1.951–3.618)	1.71 (1.256–2.319)
Community media exposure				
Low (R)			1	
High		–	1.42 (1.029–1.964)	–
Community ANC utilization				
Low (R)			1	1
High		–	2.54 (1.842–3.492)	1.55 (1.132–2.127)

Random effect	Model I	Model II	Model III	Model IV
Community variance (SE)	10.246* (1.020)	1.118* (0.179)	0.773* (0.134)	0.613* (0.128)
ICC (%)	75.6	25.3	19.1	15.7
PCV (%)	Reference	89.1	92.4	94.1

Model fitness	Model I	Model II	Model III	Model IV
Log likelihood	−2285.8943	−1760.6829	−1886.9685	−1683.9758
AIC	4575.789	3557.366	3809.937	3425.952

In this table, the odds ratios were adjusted for all other variables constant in the respective models

* Significant at P-value <0.05

Model IV, the final model included both the individual and community level characteristics simultaneously. After controlling for other individual and community level factors, women who had primary education were 50 % (AOR = 1.50; 95 % CI 1.202–1.880) and women who had secondary education and above were 3.6 times (AOR = 3.60; 95 % CI 2.491–5.214) more likely to give birth at health institutions as compared to women who had no education. Regarding media exposure, women who had media exposure were 39 % more likely (AOR = 1.39; 95 % CI 1.115–1.752) to give birth at health institutions compared to women who had no media exposure. After holding other factors constant, women from richest households had 74 % higher (AOR = 1.74; 95 % CI 1.143–2.648) odds of institutional delivery as compared to women from poorest households. Looking at birth order, women with birth order of two to three were 51 % (AOR = 0.49; 95 % CI 0.385–0.622); women with birth order of four to five were 62 % (AOR = 0.38; 95 % CI 0.286–0.512) and women with birth order of six and above were 77 % (AOR = 0.33; 95 % CI 0.245–0.456) less likely to give birth at health institutions compared to women who had first order births. Women who had one ante natal care visit were 89 % (AOR = 1.89; 95 % CI 1.148–3.113) and woman who had two to three antenatal care visits were 2.7 times (AOR = 2.66; 95 % CI 2.031–3.479) more likely to give birth at health institutions compared to woman who had no antenatal care checkups. Similarly, women who had four and above antenatal care visits were 4.4 times more likely (AOR = 4.43; 95 % CI 3.405–5.751) to give birth at health institutions compared to women who had no antenatal care checkups.

Keeping other variables constant, women from urban areas were almost 4.7 times more likely (AOR = 4.74; 95 % CI 3.196–7.039) to give birth at health institutions compared to their rural counterparts. Women residing in communities with high proportion of educated women had 71 % higher (AOR = 1.71; 95 % CI 1.256–2.319) chance of institutional delivery as compared to women residing in communities with low proportion of educated women. Similarly, women residing in communities with high ANC utilization rate were 55 % more likely (AOR = 1.55; 95 % CI 1.132–2.127) to give birth at health institutions than women residing in communities with low ANC utilization rate.

After the inclusion of both the individual and community level variables in model IV, the variation in the odds of institutional delivery care between communities still remained statistically significant(τ = 0.613, p-value = 0.000). As shown by the estimated ICC, 15.7 % of the variability in institutional delivery service utilization was attributable to differences between communities. The PCV indicated that, 94.1 % of the variation in institutional delivery service utilization across communities was explained by both individual and community level factors included in model IV.

## Discussion

This study was based on the data of 2011 Demographic and Health Survey conducted in Ethiopia. The study has identified several factors that have significant influence on the utilization of health institutions for child birth. The finding of this study showed that women education exerts a positive significant influence on the use of delivery care services. This result concurred with findings of several studies [14–22]. The possible explanation could be, educated women have a greater confidence and capabilities to take actions regarding their own health and have the ability and willingness to travel outside home to seek out modern and quality health care services. In addition, educated women have greater exposure in accessing relevant health information on maternal health services thus enabling them to seek proper medical care whenever necessary.

Results of this study verified that women from richest households had higher odds of institutional delivery than women from poorest households which corroborates the findings that have been reported in prior studies [10, 15–17, 31, 32]. The possible explanation could be related to the implicit costs needed to access health care services. Media exposure also affects institutional delivery positively which is consistent with the findings of other studies [33–35]. Another study in Ethiopia also indicated that knowledge of mothers on pregnancy and delivery services has a significant influence on institutional delivery [20]. This shows that access to health related information has a strong influence on institutional delivery. Literatures also documented that exposure to media is an important source for health information [12, 36] and promotes health related behavior of the women.

Pertaining to birth order, the findings of this study depicted that the odds of utilizing health institutions for delivery care decreases with an increase in child birth order. This corroborates with the findings of several studies [33–39]. A possible explanation is that, after the uneventful birth of the first child at home, subsequent deliveries are perceived to be of low risk, thus increasing the likelihood of delivering subsequent babies at home. Also, a higher birth order suggests a greater number of children in the house hold as a result of which the women might have greater responsibilities and less time to visit health facilities for delivery care.

This study also revealed that women who had at least one ANC visit for their recent birth have higher chance of institutional delivery than women who have no ANC visits. Previous studies [18–21, 32, 40–43] also reported that ANC attendance increases the likelihood of institutional delivery. ANC attendance could be a marker of familiarity of women to maternal health services. Analysis of DHS data from six African countries and a study in India have also shown that the characteristics that predispose women to seek pregnancy care also make them more likely to seek care during delivery [11, 37]. In addition, antenatal care could also be an opportunity for health workers to provide health information and to discuss on the women’s place of delivery. In a study conducted in Tanzania, women informed about pregnancy complications during antenatal care were found to be more likely to deliver at a health facility [44]. This shows that the information given to pregnant women during antenatal care is vital to promote institutional delivery service.

In this study, geographical region where a woman resides was found to be an important predictor of institutional delivery. Other studies conducted in developing countries also pointed out the significant regional variations in the use of health facilities for delivery care [15–17, 39, 45–47]. Urban residence was also found to have a positive significant association with institutional delivery. This result is in agreement with studies conducted in developing countries [24, 36, 39, 41, 48–51]. The importance of place of residence in determining women’s use of health institutions for child birth can be explained through the availability of health services. As explained by another study, urban women in Ethiopia tend to benefit from increased knowledge and access to maternal health services [10]. Another study also indicated that rural areas generally have poor infrastructure, fewer health facilities and inadequate health services compared to urban areas, making women from rural areas less likely to utilize health facilities for delivery care [12]. Moreover, women from urban areas might have higher receptivity to new health related information and familiar with modern health care.

In this study distance to health facility was negatively associated with institutional delivery service utilization. A study conducted in Tanzania also found similar results where longer distance to health facility was related to home delivery [44]. This finding is also in agreement with the results of several studies in developing countries where physical proximity to health facilities plays an important role in the utilization of delivery services [40, 52–59]. The effect of distance on the use of health services has been attributed to the time and cost of travel and poor road conditions which reduces health seeking behavior and become an actual obstacle to access health care after an individual has decided to seek care [54, 60].

This study also found that community women’s education increases the odds of institutional delivery which is similar with a study done in six African countries [11]. Women residing in communities with high antenatal care utilization rate were also found to have higher chance of institutional delivery than women residing in communities with low rate of antenatal care utilization. This finding corroborates with a study in Congo [16]. The high ANC utilization rate at community level may reflect the familiarity of the community about maternal health services and the health service use habits of women in the community which plays an important role in influencing other women’s health seeking behavior positively. Data were not present to measure the actual presence of health services, so this variable may be acting as a proxy for service availability. As a result, higher ANC utilization at the community level might show the availability of maternal health services particularly delivery services in the community.

As hypothesized, results of this study showed that community level random intercepts (variances) were large and statistically significant indicating considerable differences between communities in the propensity of women’s use of health institutions for delivery services. This supports the application of multilevel modeling for this particular study [26–29].

This study also indicated the presence of significant unobserved variations between communities beyond the influence of the measured individual and community factors. Studies conducted in Nigeria, Congo, Indonesia and six African countries also found similar findings with a significant unobserved variability in the odds of institutional delivery across communities [11, 15–17]. The unobserved effects might represent the differences among communities in terms of social norms, cultural beliefs and health service related factors like quality of health services which influences people’s attitudes and opinions towards delivery care services.

### Strengths and limitations of the study

The study was based on the most recent EDHS with a nationally representative large sample size. In addition, this study applied multilevel modeling to accommodate the hierarchical nature of the EDHS data. Despite the above strengths, the study has the following limitations. There might be recall bias given that the events took place 5 years preceding the survey. The data could have been more useful to this study if some particular information on service related factors like service availability and quality of health services had been collected.

## Conclusion and recommendations

In this study both the individual and community level characteristics were found to have significant influence on institutional delivery. Women’s education, household socio economic level, media exposure, birth order and ante natal care visit were the factors that influence institutional delivery at the individual level. The study also showed that the communities in which the women reside play a significant role in shaping a women’s decision to utilize health institutions for delivery services. Among the community characteristics place of residence, region where the women reside, distance to health facility, community women’s education and community ANC utilization rate were the factors found to be significantly associated with institutional delivery. Further researches of these factors are needed to better understand how these factors may affect the decision to seek institutional delivery.

## Abbreviations

ANC: ante natal care
AOR: adjusted odds ratio
CSA: Central Statistical Agency
EDHS: Ethiopian Demographic and Health Survey
FMOH: Federal Ministry of Health
ICC: intra class correlation coefficient
MM: maternal mortality
PCV: proportional change in variance
UNFPA: United Nation Fund for Population Affairs
UNICEF: United Nations Children’s Fund
WHO: World Health Organization
